# Opioid and analgesic utilization in Ireland in 2000 and 2015: A repeated cross‐sectional study

**DOI:** 10.1002/prp2.899

**Published:** 2021-12-16

**Authors:** Frank Moriarty, Kathleen Bennett, Tom Fahey

**Affiliations:** ^1^ HRB Centre for Primary Care Research RCSI University of Medicine and Health Sciences Dublin Ireland; ^2^ School of Pharmacy and Biomolecular Sciences RCSI University of Medicine and Health Sciences Dublin Ireland; ^3^ Data Science Centre RCSI University of Medicine and Health Sciences Dublin Ireland

**Keywords:** analgesics, drug utilization, opioids, pharmacoepidemiology, pregabalin

## Abstract

In recent decades, opioid use has increased internationally and is a major public health concern. This study aims to characterize changes in opioid and other analgesic prescribing in Ireland over a 15‐year period (2000–2015). This is a repeated cross‐sectional study of administrative pharmacy claims data in 2000 and 2015. Individuals of all ages in Ireland's Eastern Health Board region who were eligible for the General Medical Services (GMS) scheme were included. This scheme covers 40% of the population, mostly those on lower incomes and older people. The primary outcome was dispensing of opioids, both prevalence of any use and rate per 1000 GMS eligible population (standardized to the 2015 population). Logistic regression was used to assess odds of opioid dispensing in 2015 versus 2000, controlling for demographic differences. The eligible study population was 364 436 in 2000 and 523 653 in 2015. In 2000, 19.4% of the eligible population had at least one opioid dispensing compared to 20.8% in 2015. The rate increased from 671 to 1098 dispensings per 1000 population. The increase was highest in the dispensing rates of codeine, tramadol, oxycodone, buprenorphine, and fentanyl. Compared to 2000, there was higher odds in 2015 of being dispensed a strong opioid (adjusted odds ratio 2.0, 95%CI 1.97–2.04) or long‐acting formulation (3.75, 95%CI 3.58–3.92). Increased prescribing of opioids, particularly strong opioids, between 2000 and 2015 is evident in Ireland. This is concerning due to the potential for misuse, and opioid‐related morbidity/mortality.

## Graphical abstract

This repeated cross‐sectional study examined opioid and analgesic utilisation in Ireland in 2000 and 2015. Over this 15‐year period, the rate of opioid dispensing increased by 64%.

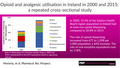

**Abbreviations**
aOR: adjusted odds ratios
aRR: adjusted rate ratios
ATC: Anatomical Therapeutic Chemical
CI: confidence intervals
EHB: Eastern Health Board
GMS: General Medical Services
HSE‐PCRS: Health Service Executive Primary Care Reimbursement Service
OME: Oral Morphine Equivalents
STROBE: Strengthening the Reporting of Observational Studies in Epidemiology
WHO: World Health Organisation

**What is already known on this subject?**

- Opioid‐related morbidity and mortality are a significant public health concern internationally, partly driven by the prescription of opioid medications.
- Other analgesic medications, such as gabapentinoids, have also been implicated in drug‐related deaths.
- To date, there is limited evidence on trends in prescription of opioids and other analgesics in Ireland.

**What this study adds?**

- Between 2000 and 2015, the prescription of opioids has increased in Ireland, with particularly sharp increases in the prescription of strong opioids and long‐acting formulations.
- Wider availability of prescribed opioids to address pain needs to be balanced against known medication‐related harms.

## 1 INTRODUCTION

Opioids have been a mainstay of malignant pain management since the World Health Organisation (WHO) analgesic ladder was published in 1986, where previously these drugs had not been prescribed widely due to concerns over potential dependence. Internationally, the use of these medications has been steadily rising, along with associated morbidity and mortality, to the extent that in the United States this has been termed the opioid epidemic.^1^

Although opioids are a mainstay in treating acute and cancer‐related pain, they are increasingly being used, and now predominantly so, for chronic non‐cancer pain where benefits are less likely to outweigh risks.^2^ A recent US study found opioid‐attributable deaths has increased threefold from 2001 to 2016, equivalent to 1.68 million person‐years of life lost in 2016.^3^ In England, prescribing of morphine‐equivalent opioids increased by 127% between 1998 and 2016.^4^ In Ireland, although codeine prescribing has been examined,^5^ there has been little evidence on trends in opioid prescribing. However, two recent studies have suggested Ireland has relatively high opioid overdose mortality, and that prescribing of strong opioids has increased between 2010 and 2019.^6^, ^7^ With changing opioids prescribing, it is important to understand if changes are driven by higher prescribing rates or doses/potencies, and also how this fits within the context of other analgesic medications. The potential for inappropriate use and misuse of analgesics is not limited to opioids, with treatments for neuropathic pain, such as pregabalin and gabapentin, also of concern.^8^, ^9^ This has resulted in the reclassification of pregabalin as a controlled drug in the UK in 2019 to reduce potential misuse and abuse.^9^ Lastly, concerns have been raised that prescribing of lidocaine patches, a recently developed analgesic product approved for post‐herpetic neuralgia, outside of their approved indication represents low‐value care based on cost and lack of robust evidence of effectiveness.^10^

Therefore, this study aims to characterize changes in analgesic, and specifically opioid, prescribing in Ireland over a 15‐year period from 2000 to 2015.

## 2 MATERIALS AND METHODS

This is a repeated cross‐sectional study based on secondary analysis of administrative dispensing data. The Strengthening the Reporting of Observational Studies in Epidemiology (STROBE) statement was used to guide the reporting of this manuscript.^11^ The setting is primary care in the Eastern Health Board (EHB) region of Ireland, which is the largest of eight regions including 29.3% of the national population. The study focuses on 2 years, 2000 and 2015, being the earliest and latest full years for which data were available when data analysis commenced in 2017, and we did not have access to data for intervening years. Data used in this study were provided by the Health Service Executive Primary Care Reimbursement Service (HSE‐PCRS) at this time. No formal mechanism of access to item‐level data exists currently. Ireland has a mixed public private health system, with approximately 40% of the population having entitlement to a range of public health services, including medicines, at low or no cost through the General Medical Services (GMS) scheme.

This study includes individuals of all ages in the EHB region of Ireland eligible for the GMS scheme. This public health scheme provides free health services (including medications, although with a small co‐payment of €2.50 per item applying in 2015) for those eligible based largely on income and age, and over‐represents socioeconomically deprived and older people. Eligibility criteria for the GMS scheme were similar in both study years, with the main difference being an end to automatic entitlement for those aged ≥70 years in 2009, however, approximately 83% of this age group were still eligible in 2015. Data were analyzed on dispensing of analgesic medications on the GMS scheme, identified using WHO Anatomical Therapeutic Chemical (ATC) codes (Table S1). This did not include methadone or specific buprenorphine formulations dispensed as opioid substitute treatment which is covered under a separate scheme.

The primary outcome was dispensing of opioid medications, expressed as both the prevalence of any use and rate of dispensings per 1000 GMS eligible population. This population was based on numbers of individuals registered with the PCRS under the GMS scheme within each study year, regardless of whether they were dispensed medication in that year. Each of these was standardized to the 2015 GMS population based on age group (<5, 5–15, 16–44, 45–64 and ≥65 years) and sex. Similarly, we also summarized the prevalence and rate of dispensing of other analgesic classes/agents (NSAIDs, paracetamol monotherapy, lidocaine patches, gabapentinoids, triptans). Opioid‐paracetamol combinations were classified and analyzed based on their opioid ingredient. Although other medications can be used in the treatment of pain, the research team judged that the above drug could be reliably assumed to be prescribed for analgesia, rather than alternative indications.

Opioid medications were further examined in terms of strong opioids (as per the British National Formulary Classification, see Table S1), long‐acting formulations (patches or modified‐release tablets), and individual opioid drugs. These were summarized in terms of rate of dispensings and Oral Morphine Equivalents (OME) per 1000 population, also standardized to the 2015 GMS population. OMEs for each dispensing were calculated by the product of the quantity, strength, and conversion factor.^4^

Logistic regression was used to determine the odds of an opioid dispensing in 2015 compared to 2000, controlling for age group and sex, as well as the odds of a strong or long‐acting formulation opioid dispensing, yielding adjusted odds ratios (aOR) with 95% confidence intervals (CI). Analysis was at the individual level, including an observation for each GMS‐eligible individual. The outcome variables were binary indicators of any dispensing of an opioid, strong opioid, and long‐acting formulation. Among those with an opioid dispensing, multivariate negative binomial regression was used to assess change in the rate of dispensings and OMEs between 2000 and 2015, yielding adjusted rate ratios (aRR) and 95% CIs. Statistical analysis was conducted using Stata version 14, and statistical significance was assumed at *p* < .05, and analytical code is available at www.doi.org/10.5281/zenodo.5570200.

## 3 RESULTS

The eligible study population ranged from 364 436 in 2000 to 523 653 in 2015. The distribution of individuals across age groups was similar for both years, with 16–44 years being the largest age group (32.1% in 2000 and 33.1% in 2015), and the percentage of females decreased from 58.7% to 55% (Table S2). The proportion of individuals receiving a dispensing of any analgesic, and each analgesic class/agent increased between 2000 and 2015 (Table 1). In 2000, 19.4% had an opioid dispensed compared to 20.8% in 2015. Similarly, the rate of dispensings also increased for analgesics and individual classes/agents (Table 1, Figure S1), with the rate for opioids rising from 671 to 1098 dispensings per 1000 population. Notably, there were substantial increases from 2000 to 2015 in the prevalence of use and rate of dispensing of lidocaine patches (from 1 to 139 dispensings per 1000 population) and pregabalin (from 0 to 324 dispensings per 1000 population).

**TABLE 1:** Standardized prevalence and rate of analgesic class/agent dispensing for 2000 and 2015 among the GMS eligible population

Class/agent	Prevalence of use (95% CI)		Rate of dispensings per 1000 population (95% CI)		% of analgesic dispensings	
	2000	2015	2000	2015	2000	2015
Analgesic (any of the below classes/agents)	44.1 (43.9, 44.2)	47.7 (47.6, 47.8)	1855 (1851, 1859)	3728 (3725, 3731)		
Opioid	19.4 (19.3, 19.6)	20.8 (20.7, 20.9)	671 (668, 673)	1098 (1096, 1101)	36.2%	29.5%
Strong opioids	4.7 (4.6, 4.7)	8.7 (8.6, 8.7)	137 (136, 139)	480 (478, 482)	—	—
Long‐acting	0.6 (0.6, 0.7)	2.2 (2.2, 2.3)	27 (26, 27)	153 (152, 153)	—	—
NSAID	32.6 (32.5, 32.8)	33.8 (33.6, 33.9)	984 (981, 987)	1183 (1181, 1186)	53.0%	31.7%
Paracetamol^a^	7.2 (7.1, 7.3)	21.0 (20.9, 21.1)	167 (166, 169)	862 (859, 864)	9.0%	23.1%
Lidocaine	0.1 (0.1, 0.1)	3.4 (3.3, 3.4)	1 (1, 1)	139 (138, 140)	0.1%	3.7%
Gabapentin	0.4 (0.3, 0.4)	1.0 (0.9, 1.0)	17 (16, 17)	70 (69, 71)	0.9%	1.9%
Pregabalin	0.0 (0.0, 0.0)	4.3 (4.2, 4.3)	0 (0, 0)	324 (323, 325)	0.0%	8.7%
Triptans	0.5 (0.5, 0.5)	1.1 (1.1, 1.2)	15 (15, 16)	51 (51, 52)	0.8%	1.4%

^a^ Relates to paracetamol monotherapy, combinations with opioids were classified and analyzed based on their opioid ingredient.

**FIGURE 1:**
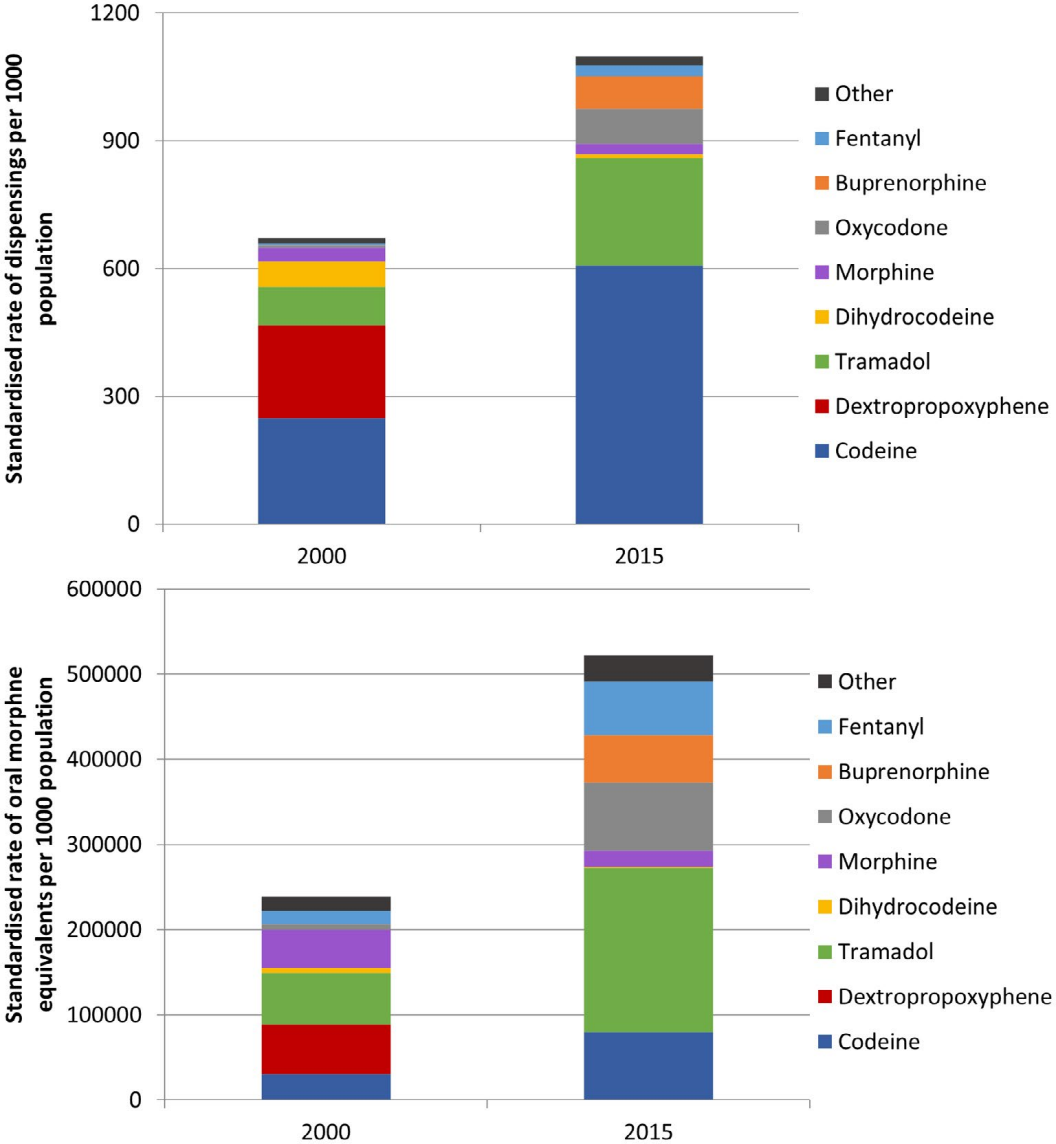
Rate of dispensings (top) and oral morphine equivalents (bottom) for individual opioids drugs in 2000 and 2015, standardized to the 2015 population based on age group and sex. Other groups include hydromorphone, pethidine, pentazocine, dextromoramide, meptazinol, and tapentadol (all <15 dispensings per 1000 during both study years)

Focusing specifically on opioids, strong opioids composed 20.4% of opioids dispensings in 2000 (137 dispensings per 1000 population) compared to 43.7% in 2015 (480 per 1000 population), while long‐acting opioids also increased (4.0% to 13.9%). Accounting for opioid strength and quantity per dispensing, there were 239 263 OMEs dispensed per 1000 population in 2000 versus 522 624 in 2015 (Table S3). For strong opioids, the rate of OMEs increased from 141 721 per 1000 population in 2000 (accounting for 59.2% of OMEs) to 440 448 per 1000 population in 2015 (84.3% of OMEs). Across both rate of dispensing and OMEs (Figure 1, Table S3), there was the greatest growth in the rates of dispensing of codeine, tramadol, oxycodone, buprenorphine, and fentanyl, while morphine, dihydrocodeine, and dextroproxyphene dispensing decreased.

The odds of an opioid dispensing was marginally higher for an individual in 2015 compared to 2000 (adjusted odds ratio 1.1, 95% CI 1.09, 1.12), however, there was a doubling in the odds of being dispensed a strong opioid (aOR 2.0, 95% CI 1.97, 2.04) and almost four‐fold increase in odds for long‐acting formulations (aOR 3.75, 95% CI 3.58, 3.92). Among those who received an opioid dispensing, the adjusted rate ratios indicated a higher rate of dispensings (aRR 1.51, 95% CI 1.50, 1.53) and rate of OMEs (aRR 1.98, 95% CI 1.95, 2.01) in 2015 compared to 2000.

## 4 DISCUSSION

Our study found increases in dispensing of all analgesic classes studied from 2000 to 2015, including increases in the prevalence and rate of opioid dispensings. There were notable increases in lidocaine patches and pregabalin, from very low levels to 3.7% and 8.7% of analgesic dispensings in 2015. Further examination of opioid use illustrated higher growth in OMEs than in opioid dispensings, partly due to greater proportional growth in some higher potency opioid agents, for example, oxycodone, fentanyl, and buprenorphine. Although the largest increase in the rate of dispensings was for codeine, it accounted for less of an increase in OMEs given its low potency. Decreasing use was observed for dextropropoxyphene, the opioid ingredient in co‐proxamol which was withdrawn from the Irish market in 2006, as well as morphine and dihydrocodeine. The decline in morphine concurrent with growth in newer opioids has been reported in other studies, as prescribers switch to these as the strong opioid of choice.^4^, ^6^, ^12^ Promotion and marketing of newer opioids and opioids formulations may have contributed to uptake of these agents.^13^

### 4.1 Comparison with the literature

Although we could not assess analgesic indication or appropriateness of prescribing, this study's findings can be compared to the extensive literature on temporal trends in analgesic utilization. Increasing use of lidocaine patches outside of their indication (i.e. for other types of neuralgia or other forms of pain) in England and Ireland has been targeted as low‐value care, prompting different approaches to cost containment in both countries.^10^, ^14^ Our findings of growth in gabapentinoid use (10‐fold and 20‐fold increase in prevalence and dispensings) exceeds those reported internationally, such as a 3‐fold increase in prevalence in the US, and a 16‐fold increase in gabapentinoid prescriptions in Scotland.^15^, ^16^

Our findings showed a 64% increase in opioid dispensings and a 118% increase in OMEs. A recent Irish study of strong opioids showed increases continued from 2015 to 2019, particularly for oxycodone and tapentadol.^6^ By comparison, research in England found items per 1000 population increased from 578 in 2000 to 762 in 2015 (31% increase), and a 115% increase in OMEs (199 000–428 000).^4^ Other international estimates include 464 000 OMEs per 1000 population in British Columbia, Canada,^12^ and 543 400 per 1000 population in the United States,^17^ although with noted variation between provinces/states. While our findings are higher than many of these, this may reflect that the GMS scheme over‐represents socioeconomically deprived and older individuals, among who painful conditions may be more prevalent.^18^

### 4.2 Strengths and limitations

This study was limited to dispensing of medicines prescribed to GMS eligible individuals, who are not representative of the total Irish population. However, these individuals are covered by state healthcare and are, therefore, the group where there is the greatest scope to optimize medication use. We could only control for limited demographic factors, and lacked information on the indication for analgesic treatment, and therefore some growth may be explained by appropriate use, for example, in the palliative setting. However, palliative care guidance in Ireland recommends oral morphine, hydromorphone, oxycodone, and codeine may be used first line, which suggests appropriate use would not account for increases across all opioids. Only prescribed and dispensed medications were included in our data source, so non‐prescription analgesic use could not be assessed. However, only a limited range of analgesics are available without prescription, and use is likely to be low among GMS eligible patients who can obtain prescriptions medicines at no or low cost. A strength of this study is that it provides new evidence on changes between 2000 and 2015 in analgesic, in particular opioid, use in a general primary care population. Although more recent data than 2015 on strong opioids have been analyzed elsewhere,^6^ our study provides an earlier historical benchmark to understand analgesic prescribing changes. It also characterizes opioid utilization using morphine equivalents, a robust measure to evaluate shifts in the quantity and potency of opioid prescribing.^19^

## 5 CONCLUSIONS

Increasing prescription use of opioids, particularly strong opioids and long‐acting formulations, and gabapentinoids is concerning due to the potential for misuse, diversion and opioid‐related death. Such concerns are reflected in reports of increasing drug‐related deaths in Ireland.^20^ However, restrictions to reduce drug‐related harm must be balanced against the need to ensure access to appropriate pain relief. Future research should examine the trajectory of opioid dispensing trends in Ireland, and how this relates to medication‐related morbidity and mortality.

## DISCLOSURE

None declared.

## AUTHOR CONTRIBUTIONS

Frank Moriarty, Kathleen Bennett and Tom Fahey conceived and designed the study. Kathleen Bennett acquired the data. Frank Moriarty conducted the analysis and Frank Moriarty, Kathleen Bennett, and Tom Fahey interpreted the data. Frank Moriarty drafted the manuscript and all authors revised it critically for important intellectual content.

## ETHICS APPROVAL

This study used anonymized data from pharmacy claims and therefore did not require ethical approval.

## Supporting information

Supplementary Material

## Data Availability

Data on pharmacy claims are held by the Health Service Executive Primary Care Reimbursement Service. Code relating to this study is available at https://doi.org/10.5281/zenodo.5570199.
