# Supplementary material for: Opioid and analgesic utilization in Ireland in 2000 and 2015: A repeated cross‐sectional study
Source: Pharmacol Res Perspect. 2021 Dec 16;10(1):e00899. doi: 10.1002/prp2.899 (PMC8675152; doi:10.1002/prp2.899)
Supplement: Supplementary file 1 — Supplementary Material [file PRP2-10-e00899-s001.docx]

# Opioid and analgesic utilisation in Ireland in 2000 and 2015: a repeated cross-sectional study

Frank Moriarty^1,2^, Kathleen Bennett^3^, Tom Fahey^1^

1. HRB Centre for Primary Care Research, RCSI University of Medicine and Health Sciences

2. School of Pharmacy and Biomolecular Sciences, RCSI University of Medicine and Health Sciences

3. Data Science Centre, RCSI University of Medicine and Health Sciences

## Appendix

**Supplementary table 1.** Description of included analgesic drug categories, sub-categories, and individual opioids, and ATC codes used to identify these.

| **Category** | **ATC codes or included medications** |
| --- | --- |
| Opioids |  |
| Morphine | N02AA01, N02AG01, N02AA51 |
| Hydromorphone | N02AA03, N02AG04 |
| Oxycodone | N02AA05, N02AJ18, N02AJ19, N02AA55, N02AJ17 |
| Dihydrocodeine | N02AA08, N02AJ02, N02AJ03, N02AJ01, N02AA58, N02BE51* |
| Codeine | N02AJ07, N02AJ08, N02AJ09, N02AJ06, R05DA04, N02AA59, N02AA79, N02BE51* |
| Pethidine | N02AB02, N02AB52, N02AB72, N02AG03 |
| Fentanyl | N02AB03, N01AH01, N01AH51 |
| Dextropropoxyphene | N02AC04, N02AC54, N02AC74 |
| Pentazocine | N02AD01, N02AD51 |
| Dextromoramide | N02AC01 |
| Buprenorphine | N02AE01 |
| Tramadol | N02AX02, N02AJ14, N02AJ15, N02AJ13, N02AX52 |
| Meptazinol | N02AX05, N02AX91 |
| Tapentadol | N02AX06 |
| NSAID | M01A (except M01AX05, M01AX12, M01AX25, M01AX26), M02AA |
| Paracetamol | N02BE01 |
| Lidocaine | N01BB02 |
| Gabapentin | N03AX12 |
| Pregabalin | N03AX16 |
| Triptans | N02C |
| **Sub-category** |  |
| Strong opioids | Morphine, Hydromorphone, Oxycodone, Pethidine, Fentanyl, Dextromoramide, Buprenorphine, Tapentadol, Tramadol |
| Long-acting formulations | Sustained release oxycodone, hydromorphone, morphine, or oxycodone/naloxone combination tablets  Buprenorphine or fentanyl transdermal patches |

* Selected relevant products included.

**Supplementary table 2**. Distribution of GMS eligible population in the Eastern Health Board region by age group and sex in 2000 and 2015.

| **Characteristic** | **n (%)** | |
| --- | --- | --- |
|  | **2000** | **2015** |
| **Age group** |  | |
| < 5 years | 26312 (7.2) | 32350 (6.2) |
| 5-15 years | 57304 (15.7) | 89249 (17.0) |
| 16-44 years | 116903 (32.1) | 173083 (33.1) |
| 45-64 years | 69258 (19.0) | 97359 (18.6) |
| ≥ 65 years | 94659 (26.0) | 131612 (25.1) |
| **Sex** |  |  |
| Male | 150402 (41.3) | 235802 (45.0) |
| Female | 214034 (58.7) | 287851 (55.0) |
| **Total** | 364436 | 523653 |

**Supplementary table 3.** Standardised rate of dispensings and oral morphine equivalents for individual opioids for 2000 and 2015 among the GMS eligible population, sorted by standardised dispensing rate in 2015.

| Drug | Dispensings/1,000 population (95% CI) | | Oral Morphine Equivalents/1,000 population (95% CI) | |
| --- | --- | --- | --- | --- |
|  | 2000 | 2015 | 2000 | 2015 |
| Codeine | 248.0 (246.5, 249.6) | 607.4 (605.4, 609.4) | 30,420 (30,401, 30,436) | 79,760 (79,739, 79,787) |
| Tramadol | 89.2 (88.3, 90.1) | 253.0 (251.9, 254.0) | 59,893 (59,868, 59,918) | 192,531 (192,494, 192,567) |
| Oxycodone | 4.4 (4.2, 4.6) | 82.2 (81.5, 82.9) | 6,240 (6,233, 6,249) | 80,100 (80,780, 80,126) |
| Buprenorphine | 0.7 (0.6, 0.8) | 75.8 (75.1, 76.4) | 592 (590, 595) | 55,131 (55,111, 55,151) |
| Fentanyl | 4.7 (4.4, 4.9) | 26.4 (25.9, 26.8) | 15,774 (15,761, 15,787) | 64,020 (63,999, 64,042) |
| Morphine | 32.5 (32.0, 33.1) | 25.3 (24.9, 25.7) | 44,281 (44,259, 44,302) | 18,951 (18,939, 18,960) |
| Tapentadol* | 0.0 (0.0, 0.0) | 14.9 (14.5, 15.2) | 0 (0, 0) | 24,467 (24,456, 24,477) |
| Dihydrocodeine | 59.2 (58.4, 59.9) | 8.0 (7.8, 8.3) | 6,633 (6,625, 6,641) | 1,410 (1,407, 1,414) |
| Hydromorphone | 3.7 (3.5, 3.9) | 2.6 (2.5, 2.7) | 12,266 (12,256, 12,276) | 5,199 (5,193, 5,205) |
| Meptazinol | 6.3 (6.1, 6.6) | 2.6 (2.4, 2.7) | 2,042 (2,037, 2,046) | 1,003 (1,000, 1,006) |
| Pethidine | 0.5 (0.5, 0.6) | 0.2 (0.2, 0.2) | 244 (242, 245) | 48 (47, 48) |
| Dextropropoxyphene* | 220.0 (218.8, 221.2) | 0.0 (0.0, 0.0) | 58,449 (58,424, 58,473) | 0 (0, 0) |
| Dextromoramide* | 1.1 (1.0, 1.2) | 0.0 (0.0, 0.0) | 1,572 (1,568, 1,576) | 0 (0, 0) |
| Pentazocine* | 0.5 (0.4, 0.5) | 0.0 (0.0, 0.0) | 858 (855, 861) | 0 (0, 0) |

* Products not available in one study year. Years of last dextromoramide, dextroproxyphene, and pentazocine-containing products withdrawal were 2008, 2007, and 2002 respectively; year of first tapentadol-containing product authorisation was 2010


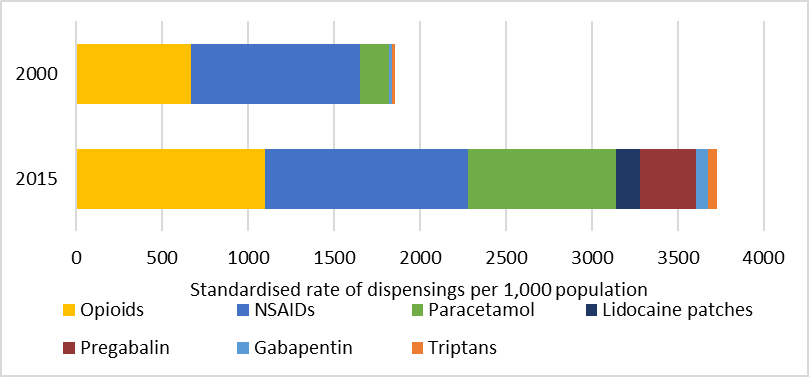


**Supplementary figure 1** Rate of dispensings for analgesic classes in 2000 and 2015, standardised to the 2015 population based on age group and sex
